# What do clinicians want? Understanding frontline addiction treatment clinicians’ preferences and priorities to improve the design of measurement-based care technology

**DOI:** 10.1186/s13722-021-00247-5

**Published:** 2021-06-15

**Authors:** Justin S. Tauscher, Eliza B. Cohn, Tascha R. Johnson, Kaylie D. Diteman, Richard K. Ries, David C. Atkins, Kevin A. Hallgren

**Affiliations:** Behavioral Research in Technology and Engineering (BRiTE) Center, Department of Psychiatry and Behavioral Sciences, University of Washington, 1959 NE Pacific Street, Box 356560, Seattle, WA 98195 USA

**Keywords:** Addiction treatment, Measurement-based care, Mechanisms of behavior change, Progress monitoring, Routine outcome monitoring, Substance use disorder treatment, User-centered design

## Abstract

**Background:**

Measurement-based care (MBC) is the practice of routinely administering standardized measures to support clinical decision-making and monitor treatment progress. Despite evidence of its effectiveness, MBC is rarely adopted in routine substance use disorder (SUD) treatment settings and little is known about the factors that may improve its adoptability in these settings. The current study gathered qualitative data from SUD treatment clinicians about their perceptions of MBC, the clinical outcomes they would most like to monitor in MBC, and suggestions for the design and implementation of MBC systems in their settings.

**Methods:**

Fifteen clinicians from one publicly-funded and two privately-funded outpatient SUD treatment clinics participated in one-on-one research interviews. Interviews focused on clinicians’ perceived benefits, drawbacks, and ideas related to implementing MBC technology into their clinical workflows. Interviews were audio recorded, transcribed, and coded to allow for thematic analysis using a mixed deductive and inductive approach. Clinicians also completed a card sorting task to rate the perceived helpfulness of routinely measuring and monitoring different treatment outcomes.

**Results:**

Clinicians reported several potential benefits of MBC, including improved patient-provider communication, client empowerment, and improved communication between clinicians. Clinicians also expressed potential drawbacks, including concerns about subjectivity in patient self-reports, limits to personalization, increased time burdens, and needing to learn to use new technologies. Clinicians generated several ideas and preferences aimed at minimizing burden of MBC, illustrating clinical changes over time, improving ease of use, and improving personalization. Numerous patient outcomes were identified as “very helpful” to track, including coping skills, social support, and motivation for change.

**Conclusions:**

MBC may be a beneficial tool for improving clinical care in SUD treatment settings. MBC tools may be particularly adoptable if they are compatible with existing workflows, help illustrate gradual and nonlinear progress in SUD treatment, measure outcomes perceived as clinically useful, accommodate multiple use cases and stakeholder groups, and are framed as an additional source of information meant to augment, rather than replace, existing practices and information sources.

**Supplementary Information:**

The online version contains supplementary material available at 10.1186/s13722-021-00247-5.

## Background

Every year, over 2.5 million American adults receive treatment in substance use disorder (SUD) facilities [1]. Even when patients receive evidence-based treatments in these settings, the clinical course and outcomes of individual patients vary considerably [2, 3].

*Measurement-based care* (MBC), which is the practice of routinely measuring and reviewing treatment progress using standardized measures, can help patients and clinicians understand whether an individual patient is responding to treatment [4, 5] and can help inform clinical decision-making regarding the current course of treatment. MBC for non-SUD mental health conditions has been associated with better treatment outcomes and improved quality of care, including better detection of clinical improvement and decline, improved therapeutic alliance, more accurate clinical judgments, and improved individualization of care [6]. However, despite the benefits of MBC for non-SUD mental health conditions, the use of MBC in SUD treatment settings has been limited [7].

Little is known about the optimal approaches for incorporating MBC into SUD treatment settings [7, 8]. Although previous research indicates that digital technologies can improve the efficiency of MBC delivery and can reduce burden to clinicians (e.g., by aiding in the administration, scoring, storage, retrieval, and display of MBC data; [9–11]), it is important to understand how MBC systems can be best designed for the clinical workflows of SUD-specific treatment settings [12]. A recent review highlighted several clinician-level barriers that impede the use of MBC in mental health treatment, including increased burden in workflows, negative attitudes toward MBC, and concerns that some outcome measures may not be relevant to patients [13]. Developing MBC systems that are perceived by SUD treatment clinicians as beneficial, minimally burdensome, and relevant to the clinical care of patients may therefore improve adoptability and implementation success, potentially leading to improved outcomes and better quality of care for patients [4–6].

The current study explored how MBC systems can be optimized for SUD treatment settings by drawing on the expertise of frontline clinicians in SUD treatment settings. We examined clinicians’ perceptions of potential benefits and drawbacks of using MBC in their clinical routines, explored their ideas for designing a MBC delivery system in the context of their existing workflows, and identified their preferences about the clinical outcomes that would be most helpful to routinely measure and monitor. We focused specifically on clinicians as key stakeholders, given their lived experiences in working intensively with patients in SUD treatment and the importance of designing an intervention that is perceived as congruent with current workflows to avoid future implementation problems [14].

## Methods

### Settings and participants

Fifteen clinicians from three SUD treatment clinics in the Pacific Northwest of the United States participated in the study. Eight clinicians were recruited from a large, publicly funded SUD treatment clinic affiliated with an academic medical center, and seven clinicians were recruited from two smaller clinics affiliated with one privately funded addiction treatment organization. SUD treatment services available at the academic medical center clinic included case management, individual- and group-based psychotherapy (outpatient and intensive outpatient), psychiatric medication management, and buprenorphine treatment for opioid use disorder. SUD treatment services at the two smaller clinics included individual- and group-based psychotherapy (outpatient and intensive outpatient). Participants were recruited via announcements in staff meetings, emails, and recruitment letters. All clinicians provided verbal consent to participate and were remunerated with $50 gift cards for their time. All procedures were approved by the University of Washington Institutional Review Board.

Demographic and professional descriptive data for the clinicians in our sample are shown in Table 1. Most clinicians were female, non-Hispanic, White, and had a bachelor’s or master’s degree. Ages ranged from 26 to 70; durations of experience working in the SUD field ranged from 6 months to 32 years. Participants reported numerous clinical approaches, with over half reporting the use of motivational interviewing and relapse prevention, and a third or more reporting case management, client-centered/humanistic, or twelve-step based approaches. Approximately a quarter or less of the sample reported using medication management, cognitive-behavioral, psychodynamic, family/couple, or other approaches.

**Table 1:** Sample Descriptive Statistics (N = 15)

	N	%
Sex		
Female	9	60
Male	6	40
Race		
White	14	93
African American	1	7
Ethnicity		
Hispanic	2	13
Non-Hispanic	12	80
Not reported	1	7
Highest degree of education		
Associate's degree	1	7
Bachelor's degree	7	47
Master's degree	6	40
Doctoral degree	1	7
Clinical approach(es)		
Motivational Interviewing	13	87
Relapse Prevention	9	60
Case Management	7	47
Client-Centered/Humanistic	7	47
Twelve-Step Based	5	33
Cognitive-Behavioral	4	27
Psychodynamic/Psychoanalytic	3	20
Medication Management	1	7
Family/Couples	1	7
Other	1	7

	Median	Range
Age	45.5	(26, 70)
Years working in SUD treatment	5	(0.5, 32)
Average caseload	30	(6, 42)

### Procedures

Each participating clinician completed a semi-structured interview with the study PI in a private setting, typically their clinic office. The interviews included questions about clinicians’ roles in the clinic, their typical work routines, and the clinical data they used to guide their treatments. The interviewer then introduced MBC and asked questions about clinicians’ perceived benefits, drawbacks, preferences, and ideas related to designing MBC systems for SUD treatment settings.

After the interviews, clinicians completed a card-sorting task in which they rated the perceived helpfulness of measuring and monitoring several indicators of clinical progress [15]. The list of progress indicators included several outcomes that are often directly associated with substance use (e.g., alcohol/drug use, craving, harms caused by substances), psychosocial functioning (e.g., depression, anxiety, employment, suicidal ideation), and hypothesized mechanisms of change that have been shown in research to often improve during SUD treatment and predict longer-term treatment outcomes (e.g., abstinence self-efficacy, coping skills, social support; [16–18]. The card sorting task only included indicators that could be measurable via patient self-report and included 23 indicators plus five blank cards for writing in additional indicators. Clinicians were asked to sort each indicator into one of three piles indicating whether the outcome would be “most helpful/always helpful”, “somewhat helpful/sometimes helpful”, or “least helpful/rarely helpful” to routinely measure and monitor in the treatments they deliver. After sorting the cards into three piles, they were asked to identify the top three most helpful indicators from the cards they rated as “most helpful/always helpful”.

After the card sorting task, clinicians completed questionnaires to report their demographics, professional backgrounds, and clinical orientations. The interview and card sorting materials are available in the Additional file 1 to this article and were developed by the research team, which included a board-certified addiction psychiatrist and two clinical psychologists with expertise in addiction treatment.

### Qualitative analysis

Interviews were audio recorded, transcribed, and loaded into QDA Miner Lite software [19] and Dedoose software [20] for mixed deductive and inductive qualitative analysis. One digital audio file for an interview was corrupted and could not be transcribed; however, card-sorting and questionnaire data from this clinician were available and included in the analysis.

In the first round of coding, a deductive coding procedure was used to identify statements in transcripts that reflected one of three a priori domains, including (1) perceived benefits of MBC, (2) perceived drawbacks or barriers of MBC, and (3) ideas and preferences related to the design and/or implementation of MBC systems within their clinical setting. Two coders developed an initial codebook with criteria for identifying instances of each domain within the transcript text. The coders then independently coded two transcripts to identify instances of these domains and reviewed their codes together to identify discrepancies and refine the criteria outlined in the codebook. The remaining transcripts were then independently coded by both coders. All coding discrepancies were jointly reviewed until 100% agreement was reached.

In the second round of coding, an inductive coding procedure was used to identify emergent themes within each of the three broader domains [21]. Two different coders first read through all transcripts, without coding, to understand overarching themes. The coders then each independently performed open coding of four transcripts (two from the public clinic and two from the private clinics) to develop an initial set of themes within the existing broader domains (benefits, drawbacks, and ideas/preferences related to MBC) and a codebook with codes summarizing those themes. Following, each coder independently applied codes to seven separate transcripts, adding new codes as they emerged from the text. Newly added codes were discussed before coding was completed on the remaining seven transcripts. All code applications and discrepancies were reviewed until 100% consensus was reached. Data was reduced (e.g., combining infrequently used codes that reflected similar themes) and findings summarized with all themes and subthemes endorsed by three or more clinicians retained and presented in the final results.

## Results

### Qualitative interviews

Qualitative interviews lasted a median of 57 min (range = 44 to 78 min). The final codebook included 11 themes and 29 subthemes. Tables 2 and 3 list the themes and subthemes that were endorsed by three or more clinicians with exemplar quotes. These themes are briefly summarized below, organized by the a priori domains of perceived benefits, perceived drawbacks or barriers, and ideas and preferences related to MBC.

**Table 2:** SUD Treatment Clinician Perceptions of Measurement-Based Care (MBC) Benefits and Drawbacks

Themes (*and subthemes*)	Example quote(s)	N clinicians^a^
Domain 1: Perceived Benefits of MBC		
Improved patient-clinician communication		
*Additional source of information (beyond patient verbal report)*	“…this report would give me more information, so it would make the client contact more…listening, less stressful …sometimes I'm…trying to get information that I need but also listen…it would be helpful to just be able to…not be so stressed about needing this information from them, and just be able to listen. I think we would both benefit from that.” “…it gave me some extra information that I didn't always glean from day-to-day interactions and things they didn't want to talk about… individuals that felt more comfortable in the writing…because of the privacy or…learning style…”	8 (4 Pu, 4 Pr)
* Helps guide in-session communication*	“If you say, ‘how'd your week go last week?’, ‘Oh, it's okay.’ That's one thing. But if we could…say, ‘Hey, I see Wednesdays, it looks like it's been a pattern of a really hard day for you. What's going on on Wednesdays?’…that could be really valuable”	8 (5 Pu, 3 Pr)
* Clinician tool to highlight change over time*	“I could see over time it being helpful, a method of tracking…two months down the road that we could look back with the patient and say, "Hey, look at the progress that you made. When you first came in you could not manage to make it to an appointment and now for the last month you've made it to every appointment on time. Or last month you told me that you were just using all the time…And now three times this last week, you were able to have a trigger and walk away from it" “…when I was doing it collaboratively with the client, I'd be like, "When you came in, you said that your depression was a 9 out of 10 and you were close to killing yourself. Today, you're saying it's a five. That's got to feel great"	7 (5 Pu, 2 Pr)
* Working as a preventative tool to notice patterns and encourage coping skills*	“….early on those withdrawal symptoms and cravings, because if we can see a pattern or know really when it's happening for people, then we can better help them determine other things to…avoid those triggers or to handle it when they are having severe cravings or withdrawals”	7 (4 Pu, 3 Pr)
* Reduces clinician bias in communication and understanding of patient*	“What I like about this is… It's just data. There's no…you're putting a positive or negative thing on it…” “I still don't want it to be like, ‘You did it. You achieved this …’ Nothing that is positive or negative. Nothing where it's like, ‘Our goal is to get to 10.’ Just more of like, ‘Is this enough of visual or a pattern for you to want to move on?’”	5 (2 Pu, 3 Pr)
Empowering to patient		
* Increased patient self-reflection*	“…[it] might be helpful to the patient…to be able to reflect on while looking at the last four months, this is what you're reporting…Were you aware of that? Develop more insight…that can be a really valuable thing for patients…” “It can be normalizing…it can encourage them…to not be dependent on outpatient services, and to realize that a lot of the work happens outside of here, and…it might feel to them like a lifeline…I'm actively doing the work”	8 (6 Pu, 2 Pr)
* Patient sees progress over time*	“Being able to see something…over time. Having the patient have access to that same information…in many ways they're more cut off from their own medical records…so they can see their progress…There's a lot of value potentially in that”	6 (4 Pu, 2 Pr)
* Patient is agent in their own care*	“…we're going to give you access to being able to see and track your own [stuff]. I think that it would be beneficial to train the client to bring awareness to their own stuff…It shows hope. There's hopefulness…that recovery's possible…” “Anything that you can track, where the person feels like they are being more of an agent in their care…”	5 (4 Pu, 1 Pr)
*Improved communication between clinicians*	“There would be this common information…people could share that would…get on the same page faster and not put the patient through so much duplication and asking questions” “…I think for our providers here too, who may be accustomed to the old style of addictions treatment, that abstinence is the only way, for me being able to go to them and say, ‘I'm asking you to write her another script and they're still using, but look, I can document this, this and this thing where they're making progress.’ It will help”	4(2 Pu, 2Pr)
Domain 2: perceived drawbacks or barriers of MBC		
Patient self-reports are subjective		
* Patient self-reports are subjective*	“…maybe you could have them doing a weekly…thing. But…they could be filling that out and… [saying that their] recovery is great, and they're taking the survey…sitting there drinking a beer…it's hard to know the validity of it”	5 (2 Pu, 3 Pr)
* Patient self-report is dependent on their mood and attitude*	“Limitations?…What problems are we going to face with [client] attitude…what's their attitude going to be like and how's that going to affect our reports that we're going to now be counting on?” “If it's completed when they come to clinic, there may be a pattern of coming to clinic that alters sort of mood and perceptions of past mood…People tend to sort of bias feelings that are more recent in their sort of assessment of how they're doing. If coming to clinic to see a provider with whom they may have, let's say, good rapport and actually this is something to look forward to in some way…”	3 (2 Pu, 1 Pr)
Lack of personalization		
* Patient may answer in a rote manner if there is not enough variation or customization of questions*	“…in our one-on-ones…we're just asking them the same questions every week… I don't know if it's a great format in following progress…Because patients tend to just say, "Yeah, yeah, yeah,"…There's no in-depth questions” “…our weekly worksheets are…the same questions every week…maybe asking those same questions in a different…way every week… so it's not like they know the answers already and…answer it…repetitively”	5 (2 Pu, 3 Pr)
* Possibility of clinicians not using reports (patient doesn’t feel heard)*	“…doing something over and over…you have to be careful it doesn't get too routine you stop thinking, because, "Oh yeah, I know this one, they're going down that road." So making a bunch of assumptions without checking in”	5 (3 Pu, 2 Pr)
* Technology is “cold” and impersonal*	“…maybe a downside could be that…the computer doesn't think like a person, so the information…would be…rigid maybe and not…thoughts or emotion going into it. It would be cold”	4 (1 Pu, 3 Pr)
Burden of time		
* Too much information for clinicians*	“…there would be some way for that information to come in…and have it be something usable…I don't want 40 lines of information every week on every patient…that's going to be real hard to find the wheat from the chaff”	4 (3 Pu, 1 Pr)
* Increased workload for clinicians*	“… [an iPad would be] another device or another thing that I would have to make sure I took care of. Obviously it would be more simple if it was somehow pulled from the stuff on the computer versus adding an iPad or something. If it's like power up this iPad and go to this app and log in … You know what I mean?”	4 (2 Pu, 2 Pr)
* Clinician anxiety or difficulty with using new technology*	“I am not very techy…have mad anxiety with techy stuff…It took me a while to learn this system…”	3 (2 Pu, 1 Pr)

^a^Pu, public organization, Pr, private organization

**Table 3:** SUD Treatment Clinicians’ Ideas and Preferences for Measurement-Based Care (MBC) Systems

Theme (*and subthemes*)	Example quote(s)	N clinicians^a^
Domain 3: ideas and preferences for MBC systems		
Minimize clinician burden		
* Support clinical documentation*	“It would be great if documentation got easier. Maybe… making it easier for [patients] to…do it on their own…when they're having an issue, explain it in their own words on some sort of domain…” “If it's something that we can just add to, like we will put notes in, if it's something that can be … some type of measurement that can be added to what we already do…just enhance what we already do.”	7 (2 Pu, 5 Pr)
* Easy integration of data with current technology*	“Then you'd have to have…a dashboard that hopefully is integrated into an [electronic medical record], God help us. That would be legible from the clinical end and actually usable that would highlight what people felt would be relevant”	7 (4 Pu, 3 Pr)
* Easy to access*	“…the number of clicks and the different places in the chart you're having to navigate. If there was one way quickly to bring this information in…” “…when I'm doing their monthly reports…I have to actually bounce back to old progress notes…to track…where they've been in the recovery process throughout the month…having that information from each progress note…generate in, so I can see throughout the month…instead of having to…go into a different area to pull that information would definitely be helpful”	5 (4 Pu, 1 Pr)
* Easy to use*	“I'm kind of task-oriented and results-oriented, so I'd really like to be able to get good results with as little effort as possible.” “So making it simple and easy to use and quite to navigate. Not a whole bunch of extra clicks.”	3 (2 Pu, 1 Pr)
* Organize information in predefined categories*	“But if this was put in there, in the weekly report like this, so I could just go skip down to this instead of go, "Strengths, S," or ‘SNAP, S for strengths’ … if it was just put in more of a format, that would be really usable for me and save me a lot of time and still being able to get some accurate information and to be able to find the information quickly”	3 (1 Pu, 2 Pr)
* Save clinician time*	“…if it was just put in more of a format, that would be really usable for me and save me a lot of time and still being able to get some accurate information and to be able to find the information quickly”	3 (1 Pu, 2 Pr)
Quantify results over time		
* Give clinician ability/option to quantifiably track patient progress*	“…a one to 10 scale on mental health. You could say, "This patient was at an eight before. They've improved to a six” “…things that…help you see patterns or…highlight consistencies over time… Over the course of a few months you might not remember… where a person's baseline was and where it is now… if there's changes or progressing”	7 (3 Pu, 4 Pr)
* Graph results*	“…if you had multiple months, you could potentially get a graph. And you're like, ‘You started off being a 1.2…and now things are 2.1, which would indicate that you seem to be feeling like you're getting better.’… You could even do an affirmation with it. ‘Hey, it feels like things are getting better’”	4 (3 Pu, 1 Pr)
* Compute an overall summary score to easily capture patient progress*	“If someone was able to come up with some sort of a score…that…combines some of these…points of data into something that provides an estimate of risk or improvement…That would be awesome”	4 (3 Pu, 1 Pr)
Easy for patients		
* Simple, short questionnaire*	“…if this thing becomes a thing that's like, ‘Ah man, I've got to answer all those questions …’ …After a while, it would actually be faster…[if] the questions are static… They might take 90 s the first week, and by the end of the month, it might take 20 s” “…only…ask this patient three questions…single sentences…bullet statements…answerable in a very clear fashion. Preferably yes or no…make it as simple as possible, because the more complicated it is, the less valuable it will be”	6 (4 Pu, 2 Pr)
* Variable preferences regarding the frequency of measurement*	“Maybe once a month…If I'm dealing with somebody with…a DUI or something, they might only be in the program for six months. You might want more frequent data that way” “Twice a week seems about right, …Monday, you capture all the crap from the weekend, and then Friday's captures all the crap…during the week. Or good stuff…too”	4 (3 Pu, 1 Pr)
* Complete as app*	“If you had an app on that that you could make a daily contact with or a weekly, or maybe even more than one. Have their daily contact app and a weekly app or something like tha”	3 (2 Pu, 1 Pr)
* Patient completes in clinic*	“…they can come in and use our computer [at the clinic] or…designated area…this is a serene room of like chill and relax…a personal space because I feel like our clients don't always have that personal space in their own homes or work or school”	3 (1 Pu, 2 Pr)
Emphasize personalization		
* Customize which measures can be utilized for different patients*	“…it's always kind of nice in terms of patients…being able to define their own goals and thinking about a treatment of monitoring or a tool. Being able to have a space to define what counts as progress for them and…that…may be different from what I consider to be the most important” “…I am usually adjusting or addressing goals and where the patient is at meeting those goals. And so I could see this being a part of that…we would definitely want something customizable and maybe a pick list type thing…or what this patient needs to work on. So we address those things first time and then can continue to readdress those as we move through treatment”	4 (3 Pu, 1 Pr)
* Use tool to help determine patient treatment placement*	“Maybe like a month in, when you want to do a treatment plan review…a month in, you've gotten to know the client a little bit. Then, you can choose what this is going to correspond to” “…I'm just not sure where our mark is going to be yet that we would decide, we've given this a really good shot and it's just not working. Let's see what else we can do for you…, determining how we can measure those would be helpful and having a system approach to it…would be helpful”	3 (3 Pu, 0 Pr)
* Option for free text and fill in responses*	“If we'd come up with the most common answers for a question, but then I'll always have another that's a free text box or things that don't fit”	3 (1 Pu, 2 Pr)

^a^Pu, public organization, Pr, private organization

### Domain 1: perceived benefits of MBC

#### Improves patient-clinician communication

One of the most frequently mentioned themes was the potential for MBC to improve patient-clinician communication during treatment, under which there were several subthemes (see Table 2). For example, clinicians expressed that MBC could provide additional data that could be compared or contrasted to patients’ verbal reports in sessions to understand patients’ experiences more fully. Several clinicians also brought up the positive influence that MBC could have in structuring their in-person sessions by helping them focus more directly on important issues that might have been raised in MBC assessment results. Clinicians additionally noted that MBC could help patients see progress over time, including changes in substance use or craving that occur gradually, nonlinearly, or that do not include complete abstinence from substance use and therefore may often go unnoticed by patients. Another perceived benefit for MBC was to help detect early warning signs (e.g., increases in craving) that could help facilitate targeted interventions to reduce the risk of adverse outcomes (e.g., unintended substance use). Finally, some clinicians observed that MBC could reduce bias in their communication with patients and thus improve their understanding of patient experiences due to the use of neutral language within standardized MBC assessments.

#### Empowers patients

Another frequently discussed theme addressed MBC potentially empowering and engaging patients more in their SUD care. Clinicians noted that MBC could encourage recognition of improvements made during treatment and greater reflection around the specific actions that patients took to achieve those improvements. Several clinicians noted that helping patients see how their outcomes changed over time could further instill self-reflection and hopefulness that change is possible, while also normalizing expectations that recovery may develop slowly or be challenged by setbacks. Many also noted that completing MBC questionnaires offered the opportunity for patients to check in with themselves during the moments they completed the questionnaires, providing additional opportunity for self-reflection, including at moments when patients may not be physically inside the treatment setting. Some clinicians noted that, collectively, these experiences could help put patients in a position of greater agency within their own recovery journey.

#### Improves communication between clinicians

A final theme highlighted that MBC could potentially improve communication between clinicians, both within and across different clinical teams. For example, some clinicians noted that MBC could provide standardized data that multiple clinicians could access for the same patient, which could reduce duplication of different providers asking similar questions to the same patient. Likewise, MBC could improve communication with providers who may have difficulty seeing patients’ improvement when abstinence has not been achieved by introducing other measures of progress capable of capturing incremental gains.

### Domain 2: perceived drawbacks or barriers of MBC

#### Patient self-report is subjective

Several clinicians expressed concerns about potential drawbacks of MBC, one of the most common being a sense of concern that patient-reported outcome measures could include unreliable, incomplete, or inaccurate information. For example, several clinicians noted that patients could self-report inaccurate information in MBC measures, for instance, reporting they are abstinent from substances or participating in recovery-related activities even if they are not. Some clinicians noted that changes in patient attitudes or moods could bias the information they self-report in MBC questionnaires, as could their rapport with clinicians and expectancies related to meeting with clinicians.

#### Lack of personalization

Some clinicians noted that MBC can lack personalization, in part because standardized questionnaires may lack context, depth, and specificity needed to fully understand a patient’s individual circumstances. Additionally, questionnaires may seem repetitive to some patients and lead to a disengaged form of responding. Some clinicians noted that they may not always be able to review the results of MBC questionnaires that patients have completed, potentially resulting in patients feeling unheard. Some providers also noted that the use of technology (e.g., computers, smartphones) to support measurement-based care could feel rigid or cold and would lack the sensitivities and emotionality of a human clinician.

#### Burden of time

Other concerns about MBC included potential increased workloads for clinicians, including time spent learning new software, managing and keeping track of hardware (e.g., tablet computers, if used), reviewing of MBC data, and addressing issues that could come to light upon reviewing MBC results. For example, reviewing MBC results may require extra time that is not currently built into clinicians’ workflows and it may take extra time to sort out which information is useful for specific patients and circumstances.

### Domain 3: ideas and preferences for MBC systems

#### Minimize clinician burden

Clinicians expressed several ideas and preferences for the design of MBC systems (Table 3), including suggestions for minimizing burden to clinicians. Suggestions included providing built-in support for clinical documentation, for example, by building features that allow MBC data to supplement or replace elements of their session notes, progress reports, or discharge summaries. Several clinicians also expressed a desire for MBC technology to be integrated with the technologies they already use (e.g., electronic health records) and to be easily accessible (e.g., minimize extra steps needed to access MBC results) to minimize workflow interruptions and more easily access clinical data from a single source. Likewise, clinicians expressed a preference for MBC systems to present the necessary clinical data in a single place (e.g., on a single summary page), in contrast to current practices of looking through multiple historical session notes to obtain necessary information to evaluate change over time. Some clinicians also expressed that organizing MBC results into predefined categories (e.g., organized into higher-level categories like “strengths” and “needs”) could make it easier to find the information they need.

#### Quantify results over time

Several clinicians expressed preferences related to how results should be quantified and displayed over time. Ideas and preferences included using simple quantitative scales that could numerically summarize clinical progress (e.g., on a 1 to 10 scale) and graphical summaries that could highlight patterns over time (e.g., improvements, decline, or stability). Some clinicians also stated a preference for a single, overall summary score that could combine multiple measures of clinical progress to efficiently understand a patient’s overall progress and/or risk for adverse outcomes.

#### Easy for patients

Clinicians expressed recommendations for making MBC systems easy for patients to use, noting that questionnaires should be short and simply worded to ensure patients could understand and remain engaged while completing them. Clinicians also reported variable preferences regarding the frequency (e.g., daily, weekly, monthly) and location that patients should complete MBC assessments. Some clinicians indicated that MBC assessments could be completed through a smartphone application, for example, to check in on clinical progress more frequently than what may be feasible if they were only completed at in-person clinic appointments. Others expressed a preference to also have an option for patients to complete MBC questionnaires in designated areas within the clinic.

#### Emphasize personalization

Finally, some clinicians expressed preferences for MBC systems to be flexible in tracking outcomes that are personalized to the needs of different patients. This could include, for example, assessing patients’ treatment goals as part of the MBC assessment process to help provide context when interpreting clinical outcome measures. Additionally, this could include an option to incorporate different outcome measures that are matched to the unique self-identified goals and concerns of each patient. Clinicians suggested considering measures that could help determine treatment placement needs. Also mentioned was a preference for including options for patients to provide narrative responses to help capture information that may not be ascertained through standardized multiple-choice questions.

### Most useful outcomes to measure in MBC

**Fig. 1:**
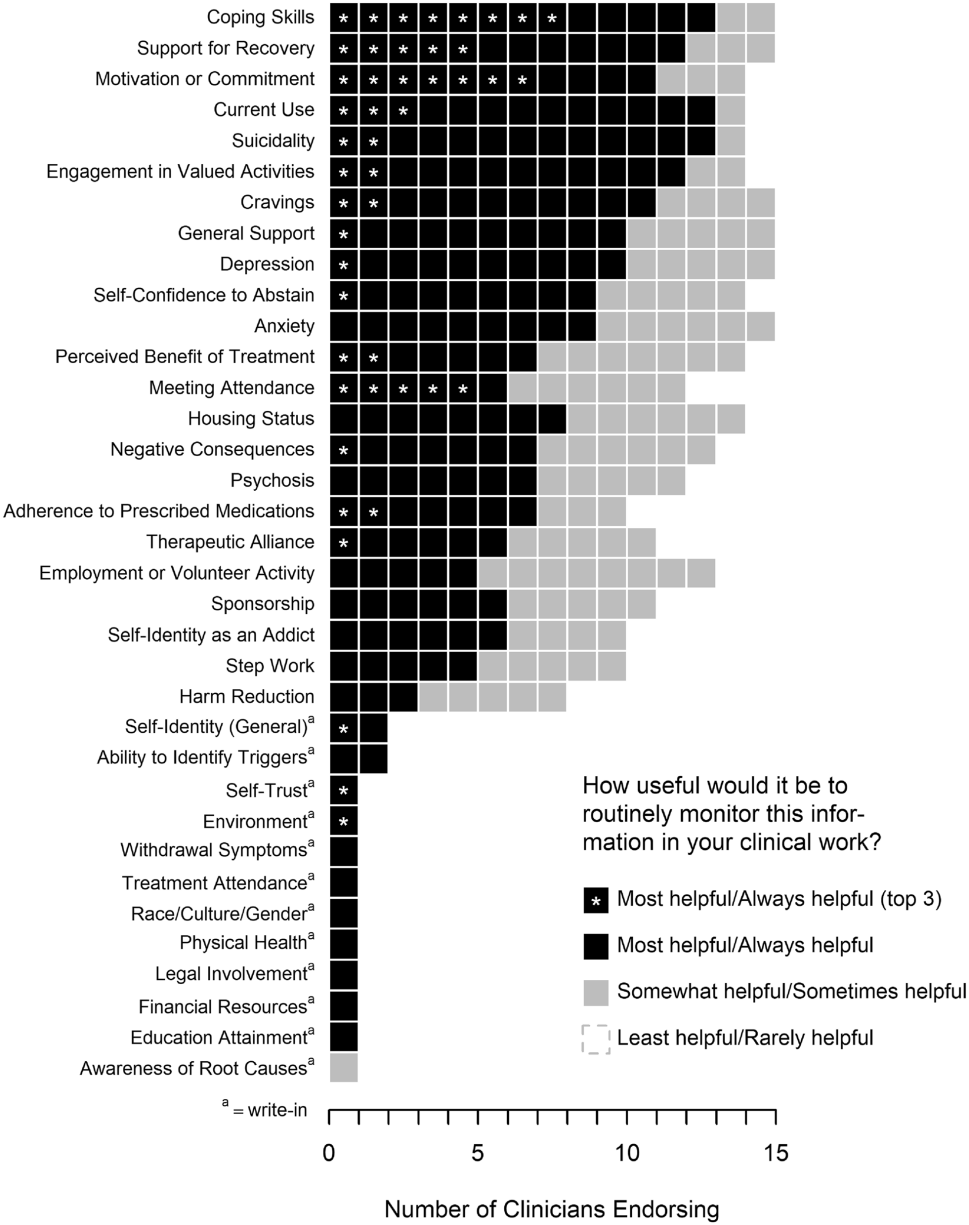
Most useful outcomes to monitor in measurement-based care, according to SUD treatment clinicians

Figure 1 summarizes the card sorting task results in which clinicians rated the usefulness of routinely measuring and monitoring various indicators of clinical progress. Twelve potential outcome measures were rated in the “most helpful/always helpful” category by the majority of clinicians; these measures spanned several broader domains related to current substance use (e.g., current alcohol and drug use, cravings), psychosocial functioning (e.g., suicidality, depression, anxiety), hypothesized mechanisms of change in SUD treatment (e.g., coping skills, motivation, self-confidence to abstain, support for recovery, general support), and other categories (e.g., engagement in valued activities, housing status).

Progress indicators that were more often rated as “somewhat helpful/sometimes helpful” or “least helpful/rarely helpful” included twelve-step work, sponsorship in twelve-step programs (e.g., Alcoholics Anonymous), self-identification as a person with addiction, employment or volunteer activities, and harm reduction outcomes.

## Discussion

Through qualitative interviews with SUD treatment clinicians, we identified potential benefits, drawbacks, ideas, and preferences related to the use of MBC in SUD treatment settings. These ideas and concerns should be considered when developing and implementing technology to support MBC in SUD treatment settings. Failure to take clinician perspectives into account may result in poor adoption of MBC tools and implementation failure [22], further perpetuating gaps in the use of MBC, an evidenced-based practice that could potentially improve the quality of care for patients in SUD treatment settings.

Clinicians identified several potential benefits of MBC, many of which are well aligned with empirically identified benefits of MBC in non-SUD mental healthcare contexts, including a better ability to detect clinical improvement and decline, improved therapeutic alliance, more accurate clinical judgments, and the ability to modify intervention plans based on assessment measures that are collected [6]. Additionally, clinicians highlighted several benefits that may be particularly salient in the context of SUD treatment. For example, clinicians noted that MBC could be helpful in allowing patients and clinicians to see treatment progress when it occurs gradually or nonlinearly, or when it does not include complete abstinence from substances. These are common experiences in the course of SUD treatment [23] and may not always indicate treatment failure [24] yet may be difficult for some patients and clinicians to notice and appreciate. Additionally, clinicians highlighted that MBC could help facilitate a more multidimensional, self-reflective, holistic, and empowering patient perspective toward SUD treatment, which aligns with contemporary conceptualizations of SUD recovery [25, 26]. This perspective diverges from the common primary focus of measuring and monitoring psychiatric symptoms in MBC for non-SUD mental health conditions [8, 9]. This perspective also contrasts common current practices in SUD treatment where clinical progress is formally or informally gauged using measures that primarily emphasize abstinence (e.g., duration of sobriety, substance toxicology test results), which may result in neglect of other forms of progress that occur outside of the context of abstinence.

The potential drawbacks of MBC that were highlighted by clinicians suggest concerns or pitfalls that may require careful attention when designing and implementing MBC in SUD treatment settings. Although these drawbacks were less frequently mentioned than potential benefits, they should be carefully addressed, including concerns about the validity of patient self-reported data, lack of personalization associated with standardized measures, workload burdens, and concerns about incorporating new technologies into workflows. Similar concerns have been described in the context of MBC in non-SUD mental health settings [13]. However, concerns about the limited validity of patient-reported measures may be particularly salient to many clinicians in the SUD treatment context, where compassion fatigue has been especially shown to engender concerns about misrepresentation and mistrust between clinicians and patients [27].

### Recommendations for MBC system design and implementation

The perspectives shared by clinicians highlight several opportunities for optimizing design and implementation of MBC systems in SUD treatment settings. Table 4 provides a list of considerations that may enhance the design of MBC technology, systems, and workflows based on the benefits, drawbacks, ideas, and preferences described by clinicians. Several overarching themes from these recommendations are briefly discussed in the text below.

**Table 4:** Summary of design considerations for MBC systems

Potential benefits	Design considerations
Improved patient-clinician communication	Design with patient-provider interaction in mind. Features could include open-ended questions that allow patients to provide additional information and context to clinicians. Design results dashboard so patients and clinicians can easily co-review and discuss MBC results and reasons for clinical changes (or barriers to change) during sessions
Empowering to patient	Ensure that displays of MBC results clearly illustrate changes in outcomes and goals over time When introducing MBC to patients, encourage them to “own” it as their own recovery-related tool, as opposed to something mandated by clinicians or clinics Support patients in accessing their own MBC data outside of clinical sessions
Improved communication between clinicians	Make MBC results accessible and understandable and relevant to clinicians from multiple disciplines who offer various types of treatments (e.g., pharmacotherapy, psychotherapy, case management, psychiatric care). Also make results understandable to clinicians who may have infrequent contact with patients and who provide non-SUD-related services. Avoid using SUD-specific jargo

Potential drawbacks	Design considerations
Patient self-reports are subjective	Emphasize to clinicians that MBC is just one additional data source, not meant to replace clinical judgment or objective measures Recognize that incentives for dishonest reporting may vary between patients and over time Emphasize that some non-substance use outcome may be less subject to bias even if there are concerns about under-reporting of substance use (e.g., coping skills, self-efficacy, depression) Consider incentivizing MBC engagement (e.g., rewards or privileges for completing assessments) and avoid punitive consequences based on responses to MBC assessments (e.g., self-reporting substance use on MBC assessments should not trigger punishment or loss of privileges)
Lack of personalization	Measure patients’ goals as part of MBC to contextualize the meaning of MBC results Measure domains that are potentially valuable across a range of patients – e.g., engagement in valued activities
Burden of time	Send MBC questionnaires automatically to patients’ mobile devices to reduce the need for clinicians to administer, score, enter, and save data from measures Make MBC results available using devices that are already available to clinicians (e.g., desktop computers) and patients (e.g., mobile devices and/or patient-facing computers in clinics, when available) Utilize existing software that does not require patients and clinicians to install and learn new software—e.g., questionnaire links that can be sent to patients via text message and completed via web browser; questionnaire results that can be viewed via web browser or electronic health record software
Clinician anxiety or difficulty with using new technology	Utilize existing devices and software to support MBC, when possible Ensure MBC data collection and storage has adequate security protections in place Communicate data security protections and limitations to patients and clinicians

Preferences and ideas for MBC	Design considerations
Minimize clinician burden	Utilize devices and software that are already used by clinicians (e.g., desktop computers, electronic health record systems, internet browsers) and patients (e.g., mobile phones, text messaging software, internet browsers)
Quantify results over time	Provide easy to read graphical summaries to summarize multiple data points Highlight or flag patients that appear to be “at risk” and in need of intervention based on historical trends in score
Easy for patients	Utilize short MBC questionnaires and collect passive data to understand how long clients spend completing measures
Emphasize personalization	Measure patient goals in addition to progress Allow SUD-specific MBC to potentially be augmented with measures for other clinical targets (e.g., depression, anxiety, insomnia) as needed

#### Develop tools to be compatible with current systems and workflows

It may be counterproductive to design MBC technology with a goal of replacing current practices or workflows for evaluating and communicating patient progress. Replacing or excessively altering current workflows, even if they are perceived as burdensome, may have unintended negative consequences on care delivery. Instead, MBC could be presented to clinicians as an additional way of obtaining information about clinical progress and goals from a different perspective than what may be currently available (e.g., verbal reports of progress by patients during treatment sessions, physical appearance, substance toxicology testing, etc.). This additional source of information is meant to supplement clinical judgement, rather than replace it. It may sometimes help with structuring and enriching clinical tasks, while also promoting patient self-reflection and empowerment.

Optimizing MBC systems for team-based care is advisable. Many SUD treatment programs rely heavily on team-based care. Ensuring that all clinicians involved in patient care have access to the same data may help clinicians who provide care to the same patient stay coordinated in their perceptions of patients’ progress and with the services they provide. Without MBC, different clinicians may each have access to different information, depending on the questions a given clinician chooses to ask when meeting with a patient or how the patient presented at that time. In contrast, implementing MBC may help all clinicians within a care team have access to a uniform set of information, while also making assessment processes more efficient and consistent across clinicians.

Integrating MBC with existing technology systems (e.g., electronic health records) may also be critical to limiting burdensomeness. Previous studies have shown that incorporating assessments into electronic health record systems facilitates the adoption of MBC [28] and incompatibility with record systems is often cited as a reason for implementation failure [29].

#### Highlight gradual, nonlinear progress with data visualization

MBC may be particularly well suited for helping patients and providers understand trends in outcomes that occur slowly, non-linearly, or imperfectly. Whereas patients and clinicians may easily notice changes that are sudden or absolute (e.g., transitioning from daily substance use to complete abstinence, or vice versa), less absolute change patterns are common in SUD treatment [23, 24] and may be more difficult for patients and clinicians to notice. Graphical displays of clinical progress over time (e.g., line graphs) may be one means of illustrating such progress. Additionally, MBC systems could potentially provide interpretations as to whether a trend reflects reliable improvement or decline, increased or decreased risk for adverse outcomes, and/or suggested interventions based on the measures collected [30].

#### Measure outcomes that are perceived as clinically useful

In contrast to common practices of gauging clinical progress based on complete abstinence from substances (or lack of abstinence), clinicians identified several indicators of change as being equally or more important than measuring substance use. Many of the outcomes that were rated as most clinically useful by clinicians reflected areas that clinicians may directly target in the psychosocial treatments they deliver (e.g., coping skills, motivation, social support) and/or represent psychosocial functioning or quality of life outcomes that patients identify as highly important (e.g., depression, anxiety, suicidality, engagement in valued activities). Including measures of some of these constructs may make MBC systems more clinically useful, while also highlighting that SUD treatment may target numerous clinical outcomes, not just substance use. This could inspire hopefulness among patients while illustrating the potential value of SUD treatment in improving multiple outcome domains that are often highly valued by patients.

#### Design to accommodate multiple use cases and stakeholder groups

MBC systems may need to be designed with flexibility to accommodate various patient and stakeholder groups. For example, patients may have differing levels of access to technology to complete MBC assessments (e.g., smartphones, computers). Additionally, patients and clinicians may vary in their comfort and ability to use these technologies. Studies suggest that most patients in SUD treatment own smartphones [31]; however, alternative, non-digital approaches to delivering MBC may be necessary for some patients who do not have computers or smartphones or who have limited skills or comfort using them. Prototypes of digital and non-digital MBC tools developed for SUD treatment settings should undergo usability testing with patients and clinicians to identify unanticipated issues with usability and understandability [15, 32].

Building in measures that are personalized to individual patients may also enhance the clinical utility of MBC. For example, MBC systems could include a core set of measures that are relevant to all patients, with options to add in additional measures that may be selected for use with different patients. Routinely assessing treatment goals and allowing open-ended responses may also help contextualize standardized progress measures and highlight the priorities of individual patients.

#### Address concerns toward patient-reported outcomes

Finally, clinician concerns about the validity of patient-reported outcomes could be addressed directly. One way to do this could be to position patient-reported outcome measures as an important but subjective form of information, meant to supplement other sources of information that may help with understanding complex clinical issues. Introducing MBC with brief training and/or case vignettes that describe how to address potentially inaccurate MBC results may help clinicians feel confident in addressing concerns about potentially inaccurate patient reports. Additionally, the benefits patients may experience by completing MBC questionnaires (e.g., self-reflection, empowerment) could be highlighted to clinicians as a potential benefit to patients, even if there are doubts about the validity of the information patients report. Moreover, treatment programs that utilize MBC could aim to develop a culture in which completion of MBC questionnaires is considered a form of treatment adherence and/or rewarded, and in which punitive clinician reactions to patients’ responses to MBC questionnaires are avoided to help encourage more frequent and candid patient responses.

## Limitations

Our study had several noteworthy limitations. First, we sampled only three SUD treatment clinics within two organizations, and it is possible that perspectives would have varied had we included clinicians from additional settings. Although our sample included providers with various training backgrounds, the size of the sample precluded us from directly comparing provider subgroups. Our sample was comprised of predominately white, non-Hispanic female-identifying clinicians and thus had limited representation of Black, Indigenous, and People of Color (BIPOC) clinicians. By design, our sample only included SUD clinicians because of their role as critical stakeholders who are vital to facilitating the use of MBC in SUD treatments; however, we did not evaluate the perspectives of other stakeholders, including patients and clinic leadership in this study. We also did not collect data on some clinician characteristics that could potentially have influenced their opinions about MBC, such as their current or historical experiences using MBC, or their personal treatment history or recovery status. Additionally, while our study identified which progress indicators clinicians perceived as potentially helpful to measure, it did not test whether those indicators were useful when measured routinely in practice, nor if they provide adequate information that would be required for MBC to be acceptable. Our interviews also focused primarily on the use of MBC for clinical practice with individual patients, as opposed to the use of aggregated MBC data across many patients or clinics (e.g., to obtain performance measures or quality indicators). Thus, there may be additional benefits and drawbacks about MBC that may not have been voiced by clinicians during our interviews (e.g., potential benefits of using aggregated MBC data to support quality improvement efforts within clinics, or potential concerns about aggregated MBC data being used to inappropriately draw de-contextualized conclusions about clinicians’ work performance [33]).

Our study also had several strengths. The data were obtained from frontline clinicians who worked in community treatment settings and offered an array of treatment approaches. The perspectives of community-based SUD clinicians have often been under-valued in SUD treatment research, despite their vast wealth of real-world experience delivering SUD treatment to diverse patient populations and extensive practice-based knowledge developed through direct clinical experience. The use of open-ended interview questions paired with rigorous qualitative analyses provided rich and detailed data, informed by real world clinical experience. Such data is critical to understanding how MBC systems may best be developed and tailored to SUD treatment settings, given the patient population and their unique experiences and needs.

## Conclusions

MBC has untapped potential for improving the quality, efficiency, and effectiveness of SUD treatment. However, efforts to implement MBC in SUD treatment settings have been hampered by a lack of research on how MBC systems should be designed to be most usable and useful in these settings. The findings of this study suggest SUD treatment providers hold positive perceptions about MBC and would likely be more welcoming of MBC systems if they are designed to be flexible, easy to use, and compatible with existing workflows. This study provides important preliminary information about how MBC technology can be designed to support these design goals and may aid the development, testing, and implementation of MBC systems that fit the unique needs of SUD treatment settings. Incorporating suggestions presented in this paper may improve MBC adoption and increase the likelihood of implementation success, ultimately leading to better a patient care experience and improved treatment outcomes.

## Supplementary Information

**Additional file 1.** Interview guide, card sort materials, and demographics questionnaire.

## Data Availability

Data sharing is not applicable to this article as only qualitative datasets containing direct quotes from clinicians were collected, which we did not obtain consent to share. Research materials used with research participants are available in supplemental materials associated with this article.

## Abbreviations

MBC: Measurement-based care
SUD: Substance use disorder
