# Supplementary material for: What do clinicians want? Understanding frontline addiction treatment clinicians’ preferences and priorities to improve the design of measurement-based care technology
Source: Addict Sci Clin Pract. 2021 Jun 15;16:38. doi: 10.1186/s13722-021-00247-5 (PMC8205211; doi:10.1186/s13722-021-00247-5)
Supplement: Supplementary file 1 — Additional file 1. Interview guide, card sort materials, and demographics questionnaire. [file 13722_2021_247_MOESM1_ESM.pdf]

Supplemental to:

What do clinicians want? Understanding frontline addiction treatment clinicians' preferences and priorities to improve measurement-based care technology designs

Contents

Semi-structured Interview Guide 2

Card Sort Materials 12

Demographics and Professional Background Questionnaire.... 17

Semi-structured Interview Guide

Consent, Confidentiality, Introduction

Introduce myself and project:

- *I'm a researcher at UW. I'm working with addiction treatment clinicians to make technology to support treatment providers working in addictions that I hope will help with monitoring patients' progress during addiction treatment.*

- *I'm interviewing people who work in the clinic to better understand your preferences, workflows, and how you do the work that you do. I'm asking for your input to understand how technology could potentially be helpful and where it could potentially cause problems.*

- *I'm especially interested in knowing how we could design something that (a) requires minimal or no additional work for clinicians, (b) tracks the patient outcomes that you're most interested in, and (c) fits with your existing workflows.*

Consent waiver and confidentiality:

- *The information you share in here is confidential. It won't be shared directly with anyone, including your colleagues and supervisors. It may be shared with other research staff (e.g., research assistants) but will remain de-identified.*

- *The findings from these interviews may be shared with other researchers and administrators, but your information will remain confidential.*

- *I'm recording these interviews because I may not always be able to write down everything that you say. However, we do not have to record this if you prefer not to. Is it alright with you if I record this interview?*

- *Explain waiver of written consent form.*

Rapport-Building and Clinical Caseload

Tell me about the kind of work you do in the clinic.

Tell me about the types of patients you usually work with – their characteristics, what they struggle with.

Tell me about your counseling approach. What kinds of issues and goals do you generally focus on in your clinical work?

What are some of the harder or more time-consuming aspects or tasks in your work? (e.g., documentation? assessments? regulations? challenging clients? heavy caseloads?)

Clinical Routine

Imagine a few typical patients you've worked with recently. When you're meeting with a typical patient on a routine basis, what kinds of things are you looking for during your sessions?

With a client you see more regularly, what things do you do <u>before</u> the session to prepare?

With a client you see more regularly, what things do you typically do <u>during</u> your treatment sessions?

With a client you see more regularly, what things do you typically do <u>after</u> a treatment session?

Do you have a template or outline that you follow to help you cover the topics you need to during your clinical sessions, or in your documentation? [Can you show me?]

Documentation Practices

Do you take notes during counseling (Y/N)? By hand or on computer? Do you complete documentation during session (Y/N)?

What goes into your note-writing? Can you tell me more about the process? What kinds of information do you usually put in the documentation?

[if not already known] What kind of patient information do you monitor in the EHR? (e.g., urine test results, clinical notes from other providers)

Can you show me (walk me through) how you would typically review a patient's chart and what kind of information you're usually looking for?

MBC-Specific Questions: General Usability and Usefulness

Give a 1-2 sentence overview of the MBC concept: As you may know, I'm hoping to explore how technology may help addiction treatment providers. One way I'm thinking of involves using technology to help monitor and track patients' progress during addiction treatment. For example, routinely collecting patient-reported outcomes in addiction-related domains via computer or iPad, and having that information instantly available to you to help you monitor treatment progress, and potentially provide information that helps you in your clinical sessions and potentially assisting with documentation requirements.

I'm curious what you think about it… what opportunities you might imagine? What things might be helpful or unhelpful? How could anything like that fit with the work that you do? Anything that seems potentially interesting to you?

[<u>If not known</u>] What could we do that would make this kind of technology helpful with your documentation or note-writing?

[<u>If not known</u>] What could we do that would make this kind of technology helpful for your sessions?

What potential problems could you see with this kind of technology?

MBC-Specific Questions: Logistics

Thinking of a few of your typical patients, how do you imagine they would feel about answering a short questionnaire before each of their clinical sessions?

What might be a good way to have these questionnaires fit with the flow of patients in the clinic? When would be best for them to complete them? How frequently? How long should it take?

How might you imagine reviewing the results of these patient questionnaires in your workflow?

When might you review them? How frequently? How long might you spend?

Technology Logistics

[Skip if fewer than 10 minutes remain]

Can you tell me about the types of technology (e.g., paper-and-pencil, desktop computer, iPad) that you think would be feasible for patients to use in the clinic? What cognitive and physical limitations might prevent some patients from using iPads or computers? What types of technology patients have access to or are comfortable with?

What worries or concerns do you think clinicians could have about using technology to track patient progress? (e.g., that we would use it to gauge their effectiveness as clinicians? Or that it might not capture the essence of SUD treatment? Or possible retribution in some way?)

MBC-Specific Questions: Content

By now you've mentioned ______ as examples of things you often monitor in your work. What other kinds of things do you monitor in your patients to get a sense of whether they're improving or staying on track during treatment?

Are there different things that you monitor to tell if a patient is failing to improve, or getting worse (as opposed to getting better)?

[CONDUCT THE CARD SORT]

Wrap Up

[<u>Administer Demographics Questionnaire</u>]

That concludes everything that I have for you today. What questions do you have for me at this time?

After I've completed all the interviews, I plan to summarize the results and present a general summary to your clinical team.

Thank you for your time and for your thoughts about this issue. I really appreciate it!

Card Sort Materials

**Current Use**<br><br>of alcohol and drugs	**Cravings**<br><br>to drink or use drugs
**Self-Confidence**<br><br>in ability to abstain from alcohol and drug use	**Coping Skills**<br><br>to help abstain from alcohol and drug use
**Motivation or Commitment**<br><br>to abstain from alcohol and drugs	**Self-Identity**<br><br>as a person with who suffers from addiction
**Meeting Attendance**<br><br>in Twelve-Step Programs	**Sponsorship**<br><br>in a Twelve-Step Program

**Step Work**<br>in a Twelve-Step Program	**Depression**<br>symptom severity
**Anxiety**<br>symptom severity	**Psychosis**<br>symptom severity
**Suicidality**	**Adherence to Prescribed Medications**<br>(psychiatric and addiction-related meds)
**General Support**<br>from close relationships	**Support for Recovery**<br>from close relationships

**Engagement in Valued Activities**	**Negative Consequences**<br>experienced from using alcohol or drugs
**Harm Reduction Strategies**<br>used while consuming alcohol or drugs	**Employment or Volunteer Activity**
**Housing Status**<br>or housing stability	**Therapeutic Alliance**
**Perceived Benefit of Treatment**	(write-in)

**Most helpful / Always helpful**	**Somewhat helpful / Sometimes helpful**
**Least helpful / Rarely helpful**	(write-in)
(write-in)	(write-in)
(write-in)	(write-in)

Demographics and Professional Background Questionnaire

These items ask about your demographic background and professional work:	
**Demographics**	
1. Gender	1 = Female<br>2 = Male<br>3 = Transgender<br>4 = (Prefer not to say)
2. Race (Circle all that apply)	1 = American Indian or Alaska Native<br>2 = Asian<br>3 = Black or African American<br>4 = Native Hawaiian or Pacific Islander<br>5 = White or Caucasian<br>6 = Other<br>7 = (Prefer not to say)
3. Ethnicity	1 = Hispanic or Latino<br>2 = Not Hispanic or Latino<br>3 = (Prefer not to say)
4. Age	________ years<br>(may leave blank if you prefer not to say)

Occupation	
5. What is your primary role(s) at your clinic?	(circle all that apply)<br>1 = Case Manager<br>2 = Certified Counselor<br>3 = Chemical Dependency Professional<br>4 = Mental Health Counselor<br>5 = Nurse<br>6 = Social Worker<br>7 = Psychiatrist<br>8 = Psychologist<br>9 = Researcher<br>10 = Administration<br>11 = Supervisor<br><br>Other: ___________________
6. Credentials (e.g., MSW, RN, LPN, etc.)	
7. Highest degree obtained	1 = High School<br>2 = Associate’s degree or professional certificate<br>3 = Bachelor’s degree<br>4 = Master’s degree<br>5 = Doctoral degree
8. Are you currently licensed in your profession?	Yes No
9. How many years have you been employed *in your current setting*?	________ years
10. How many years have you been employed as a clinical provider or administrator at *any site*?	________ years

11. How many years of alcohol or drug addiction-related experience do you have?	________ years
12. If you are an administrator, how many years have you been an administrator?	________ years

Please complete the following section only if you currently provide clinical services.

**Work Activities (clinicians only)**	
13. In a typical week, how many hours do you spend providing face-to-face clinical services of any kind?	________ hours
14. In a typical week, how many hours to you spend providing face-to-face clinical services care focusing on alcohol or drug use?	________ hours
15. In a typical week, how many hours do you spend writing clinical notes or writing other documentation?	________ hours
16. In a typical week, how many hours do you spend reviewing clinical notes or other documentation?	________ hours
17. In a typical week, how many hours do you spend on non-clinical duties (e.g., meetings, continuing education, supervision, administration, teaching)	________ hours
18. On average, how many patients do you typically have in your case load?	________ patients
19. On average, how frequently do you see new patients for routine appointments? (e.g., weekly, monthly)	

20. On average, how many new patients do you see per month?	________ patients
21. On average, how long are your clinical appointments with new patients?	________ minutes
**Clinical approach (clinicians only)**	
22. What clinical approaches do you most frequently use in your clinical work? (Circle all that apply)	1 = Case Management<br>2 = Client-Centered/Humanistic Counseling<br>3 = Cognitive-Behavioral Therapy<br>4 = Family or Couples Therapy<br>5 = Motivational Interviewing<br>6 = Twelve-Step Based Treatment<br>7 = Psychodynamic/Psychoanalytic<br>8 = Relapse Prevention<br>9 = Medication Management<br>10 = Other _________________
